# Successful Operation for Lithotripsy, in a Child Four Years of Age

**Published:** 1838-03-28

**Authors:** J. Randolph

**Affiliations:** Surgeon to the Hospital


### CLINICAL REPORTS.

### PENNSYLVANIA HOSPITAL.

*Successful Operation for Lithotripsy, in a child four years of age, by* J. Randolph, M. D., *Surgeon to the Hospital.*

The patient was a female child four years old. Previous to the operation, the sound was introduced several times to accustom the urethra to the presence of instruments. *January 18th,* Jacobson's small-sized instrument was introduced, but found to expand beyond the stone. *January 22d,* Heurteloup's intrument of the largest size was then used, and the stone was immediately twice seized, and crushed. After the operation, several large fragments of a soft stone were passed, which proved to be the phosphate of lime. Little inconvenience was experienced by the patient, except that occasionally the passage of a fragment through the urethra gave the little girl pain enough to make her complain, never, however, sufficient to confine her to bed. The hip-bath was ordered for the relief of pain, when it occurred. Since the stone was broken, she retained her water longer and suffered much less pain than previously.

*January 28th.* The stone was caught three times to-day, the instrument expanding one and a half inches upon embracing it. A slight discharge of blood followed the removal of the instrument.

*Feb. 8th.* The girl suffered for an hour severe pain in passing her urine. Hip bath.

11*th.* The stone was caught three times.

18*th.* Caught four times. A large sound was introduced to dilate the urethra.

25*th.* Caught four times.

*26th.* Passed nearly a teaspoonful of very small fragments since yesterday.

*March 3d.* Caught four times.

*14th.* Passed several fragments since the last operation. Sounded by Drs. Harris, Norris, and Randolph, who agreed that no stone could be felt. The child has not been at any time confined to bed, since the first operation, and has gone daily into the open air, during the fine weather, and has enjoyed throughout perfectly good general health. There were eight sittings in all, and at no time, did the operation occupy more than two minutes, giving the child, as she said, very little pain. Dr. Randolph operated at intervals of a week, in order to guard against any possibility of cystitis supervening.

